# The Semantic Representation of Event Information Depends on the Cue Modality: An Instance of Meaning-Based Retrieval

**DOI:** 10.1371/journal.pone.0073378

**Published:** 2013-10-28

**Authors:** Kristina Karlsson, Sverker Sikström, Johan Willander

**Affiliations:** 1 Department of Psychology, Stockholm University, Stockholm, Sweden; 2 Department of Psychology, Lund University, Lund, Sweden; 3 Center on Autobiographical Memory Research, Aarhus University, Aarhus, Denmark; University of Helsinki, Finland

## Abstract

The semantic content, or the meaning, is the essence of autobiographical memories. In comparison to previous research, which has mainly focused on the phenomenological experience and the age distribution of retrieved events, the present study provides a novel view on the retrieval of event information by quantifying the information as semantic representations. We investigated the semantic representation of sensory cued autobiographical events and studied the modality hierarchy within the multimodal retrieval cues. The experiment comprised a cued recall task, where the participants were presented with visual, auditory, olfactory or multimodal retrieval cues and asked to recall autobiographical events. The results indicated that the three different unimodal retrieval cues generate significantly different semantic representations. Further, the auditory and the visual modalities contributed the most to the semantic representation of the multimodally retrieved events. Finally, the semantic representation of the multimodal condition could be described as a combination of the three unimodal conditions. In conclusion, these results suggest that the meaning of the retrieved event information depends on the modality of the retrieval cues.

## Introduction

The semantic content (i.e., meaning) is a core feature of naturally produced language and in verbally narrated autobiographical memories (i.e., personally experienced events). The aim of the present study was to investigate the semantic representation of unimodally and multimodally cued events. For this purpose we adopted latent semantic analysis, which is a computerized method for generating semantic representations [1]. This semantic representation may be viewed as a model of a generic semantic memory in the sense that it can be used to generate semantic associations that are comparable to human associations. In the present work, we investigated whether the semantic representation of autobiographical events differed across the cue modalities. A further aim was to study the modality hierarchy within multimodal retrieval cues.

Autobiographical events can be retrieved using different retrieval methods, for example, free recall or cued recall [2]. The present study focuses on cued retrieval and utilizes the Galton-Crowitz paradigm, in which participants are asked to relate autobiographical events to retrieval cues (e.g., words, pictures, sounds, odors). Previous studies on cued retrieval of autobiographical events have mainly investigated unimodal cues (odors, pictures, sounds, words) [3]–[11]. However, in naturalistic settings individuals most often attend to and integrate sensory information pertaining to different sensory modalities simultaneously. Thus, the rationale for studying the semantic representation of event information in relation to both unimodal and multimodal retrieval cues is based on the hypothesis that retrieval cues of various modalities map differently onto stored event information (e.g., [11]). Several (unimodal) studies on cued retrieval of autobiographical events have suggested that retrieved memories differ as a function of cue modality. These studies have until now mainly been concerned with differences in the age distribution of retrieved events [4], [9], [10], [11] or in ratings of phenomenological qualities (i.e., subjective experiences such as valence and feeling of being brought back in time) [4], [7], [8], [10], [11]. In contrast the present study focuses on the content of the retrieved events and its semantic representation.

With regard to the age distribution it has been shown that autobiographical events retrieved with verbal or visual cues originate mainly from the period between 10–30 years of age [9], [10], [12]. In comparison, memories evoked by olfactory cues typically originate from early childhood (<10 years of age) [4], [5], [10], [11].

Cue modality effects have also been observed in the phenomenological experiences of recollected events [5], [7], [8], [10], [11]. Several studies have shown that olfactory evoked memories are more emotional and produce a stronger feeling of being brought back to the original event, than memories retrieved with other cue types [5], [7], [8], [10], [11]. Willander and Larsson [11] compared memories evoked by perceptual cues (i.e., odors), word cues (i.e., odor labels) or perceptual word cues (i.e., odors and odor labels in conjunction). They found that events retrieved with perceptual cues (i.e., odor-evoked memories) were rated as more emotional and associated with stronger feelings of being brought back in time than memories evoked by word cues or combination cues. Likewise, results from Cady, Harris and Knappenberg [3] suggested that memories triggered by perceptual cues (i.e., auditory or visual) produced stronger feelings of being brought back in time compared to word cues (i.e., lyrics). Further support for cue modality effects are provided by brain imaging studies, which suggest that the pattern of brain activation differ depending on the modality of the retrieval cue [14]–[15].

A central question that follows directly from these findings is how event information is selected and retrieved by different modalities. According to Conway and Pleydell-Pearce [13] autobiographical memories are dynamic and temporary constructions that are built up from information retrieved from an autobiographical knowledge base. The information in this knowledge base is organized into three levels: lifetime periods, general events and event-specific knowledge (ESK). Information higher up in this hierarchy (i.e., lifetime periods, general events) pertain more to semantic memory whereas information further down pertain more to episodic memory (i.e., ESK) [16]. The ESK is the lowest level of event information and is unique for each event. Given that the autobiographical knowledge base is a structure for organizing and storing autobiographical information, retrieval mechanisms are required in order for information from the autobiographical knowledge base to be activated and recollected. Previous studies have suggested two types of retrieval mechanisms – generative and direct retrieval [13], [17]. Generative retrieval is a form of retrieval where information is searched for whereas in direct retrieval a cue activates stored information without any prior search. More specifically, generative retrieval is a cyclic process containing three stages: specification, match and evaluation. In the first stage a description of what is to be retrieved is specified. Next, in the second stage information to be retrieved is searched for. Finally, in the third stage of this cyclic retrieval model, information is evaluated and compared to the specification. If the information does not match the specification the information is not retrieved and the search continues. However, if the information meets the specification the information is retrieved. Importantly, one form of specification could potentially be semantic relationships. Therefore, we speculate that the observed differences in retrieved events following exposure to perceptual cues could be related to the occurrence of semantic relationships specified in the description.

In comparison, direct retrieval bypasses the stages of generative retrieval. The direct retrieval works by means of a one-to-one mapping of retrieval cues and information in the autobiographical knowledge base. Direct retrieval occurs when a retrieval cue maps onto information in the autobiographical knowledge base resulting in a spread of activation emanating from the ESK which in turn results in a stable pattern of activation comprising information from all three levels [13].

An important finding concerning sensory processing is the visual dominance, which in the perceptual domain “refers to the observation that in bimodal environments vision often has an advantage over the other senses in humans” [18] (p.304). The significant role of the visual modality has been observed in both cognitive and perceptual experiments, as faster or more frequent responses to visual cues compared to other modalities [19]–[21]. Williams, Healy and Ellis [21] made a similar observation for autobiographical memory retrieval when they showed that higher ratings of vividness were related to faster response latencies and higher specificity. Their results also indicated that the second most significant modality to contribute to this effect was the auditory system.

Gärdenfors [22] suggested that semantics (i.e., meaning) could be conceived as linguistic constituents mapping onto cognitive structures. The bases of these cognitive structures are their conceptual spaces, which are spanned by quality dimensions such as color and pitch [22]. For the purpose of the present study Gärdenfors' theory provides a theoretical rationale to test if the multimodal condition could be described as a combination of the unimodal conditions. From Gärdenfors' [22] theory the following is postulated: the unimodal conditions could be conceived as the quality dimensions of a conceptual space (i.e., equivalent to base vectors spanning an n-dimensional space) and the multimodal condition a point that is potentially located in this conceptual space (that is spanned by the unimodal conditions). If the semantic representation of the multimodal condition can be described as a linear combination of the semantic representation of the unimodal conditions we expect to find the multimodal representation located within this geometric structure. In this case the geometric structure is a triangle because it is spanned by the three representations of the unimodal conditions. The triangle is formed by connecting the mean values of the unimodal conditions. Consequently this hypothesis is denoted by the triangular hypothesis.

To quantify and investigate the content of cued events we utilized latent semantic analysis (LSA). LSA is a computerized content analytic method, which can be used to measure the semantic similarity between words and passages. This is made possible by analyzing co-occurrences of words in a large corpus of text. The input to the LSA algorithm is a word-by-context frequency matrix. Cells in the matrix represent the logarithm of the frequency of occurrence of a word in a passage plus one (because log 0 is not defined). A data compression algorithm, called singular value decomposition (SVD), is applied to the resulting matrix. SVD is a mathematical decomposition algorithm, and has similarities to factor analysis and multidimensional scaling [23]–[24]. SVD decomposes the matrix so that words are re-represented as vectors in a high dimensional semantic space. The final output consists of semantic representations from which semantic scales are calculated [24]. For more descriptions of this method see [25]–[28].

Previous content analyses of retrieved events have been based on more classical strategies, such as word frequencies or manual categorization (e.g., [5], [29]–[31]). However, at least three critical issues may be identified with the classical approach to content analysis: (i) the lack of a data driven quantification of the underlying semantic representation, (ii) sensitivity to individual differences and artifacts from human evaluations in the coding/analysis process of event information, and (iii) it is labor intensive and as a result highly time consuming. Thus, in order to solve these problems, in the present study semantic representations were generated based on LSA [1] and these representations were used to study the retrieved event information.

In summary, the aim of the present study was to examine the semantic representation of event information cued by uni- and multimodal sensory information. The retrieved events were analyzed by means of semantic scales generated from the semantic representations as described below. To the best of our knowledge no previous study has quantified the semantic content of retrieved events using semantic representations.

The following three hypotheses were tested in the present study: The differential hypothesis, the visual dominance hypothesis, and the triangular hypothesis.

The differential hypothesis suggests that memories cued by visual, olfactory, auditory, and multimodal information differ in their semantic representations. This hypothesis is based on previous studies indicating differences in age distribution [4], [9], [10], [11] and ratings of phenomenological experiences [4], [7], [8], [10], [11].

The visual-dominance hypothesis suggests that the semantic representations of visually evoked events will be closer to multimodally evoked events, followed by auditory and olfactory evoked events. This hypothesis is based on the results from studies indicating visual dominance in perceptual attention [19], retrieval latency [21] and age distribution of autobiographical memories [Willander, Sikström & Karlsson, unpublished data].

In the triangular hypothesis it is tested whether the semantic representation of multimodally cued autobiographical events can be conceived as a combination of the unimodal condition or whether multimodally cued events are different from unimodally cued events. More specifically, the triangular hypothesis states that if the semantic representation of the multimodal condition is located within a geometric structure (in this case a projection from the high dimensional semantic representation to a two dimensional triangle), that is spanned by the three semantic representations of the unimodal conditions, then it can semantically be conceived as a combination of the unimodal conditions. However, if the multimodal condition is located outside of the triangle it would support the notion of supra-additive effects in multimodal retrieval. This hypothesis is based on Gärdenfors' [22] theory of conceptual spaces.

## Methods

### Ethics statement

All participants provided written informed consents prior to their participation. The project (“Multimodal processes in the retrieval of autobiographical memory”) was reviewed and approved by the Stockholm Ethical Committee (EPN #2009 417–31).

### Participants

Eighty participants (60 women and 20 men; age range 19–42 years; mean age *M* = 25.86 years, *SD* = 6.03), who were students in the Department of Psychology, Stockholm University, participated in the study for course credits.

### Design

The design was a four-way between-group design, where each participant was randomized to one of the cue conditions (i.e., visual, olfactory, auditory or multimodal). The semantic representations of the four conditions and their four associated semantic scales were used as the dependent variables.

### Materials

The stimulus materials consisted of 15 pictures, 15 sounds and 15 odors (see Appendix A). Each triad of cues represented a naturalistic setting. The unimodal conditions comprised cues from one modality whereas in the multimodal condition cues from the three modalities (i.e., visual, auditory and olfactory) were presented simultaneously. For example, participants in the unimodal conditions were presented with either the picture of an indoor swimming bath, the sound of water splashes and laughter, or the smell of chlorine. However, in the multimodal condition the picture, odor and sounds would be presented in conjunction. The visual cues were chromatic and presented on a 22-inch LCD computer screen. Sounds were presented with a pair of AKG 701 reference headphones connected to the same computer that controlled the visual presentation. Odors were kept in opaque glass jars and covered with cotton pads to prevent visual inspection. The participants held the odor jars themselves and started sniffing when given a signal.

### Procedure

All participants were tested individually. The procedure was as follows: The participants were presented with a retrieval cue and asked to retrieve an autobiographical event related to the cue. The cues were randomized for each individual and each cue was presented for a maximum of 30 seconds. In instances of successful event retrieval, the participants were first asked to write down a brief event title followed by a verbal narration of the event. Three minutes was allowed for verbal narration. After the verbal narration, participants rated each event on five phenomenological dimensions (i.e., emotionality, valence, importance, vividness and feeling of being brought back to the occurrence of the original event). Note that these ratings were collected as part of another study and are therefore not included in the present paper. If a participant was unable to recall an event for a given cue within the 30 seconds of presentation, the experiment continued with the next cue. After the retrieval phase, the participants dated the retrieved events based on their age at the time of the event. The data is available upon request.

### Analysis

The autobiographical memories were analyzed with a semantic test as described here. The analysis was made in a web based software for statistical analyzing of semantic representation, which can be found on www.semanticexcel.com. The verbally narrated memories were recorded and transcribed. The corpus of the texts (798 events; 780 Kb) was too small to construct a semantic space with a high quality of associations. Instead, the semantic space was generated from a considerably larger corpus based on a Swedish version of Google N-grams (see the *Google* n-gram project: http://ngrams.googlelabs.com). The corpus consists of approximately 1 Terabyte of text. Based on 5-grams contexts (i.e., sequences of 5 words), we created a co-occurrence matrix, where the rows in the corpus consisted of the 120000 most common words, and the columns of the 2000 most common words. Each cell represented the frequency of co-occurrence in the matrix, where words at a distance of 4 words were weighted with a factor 1, distance of 3 a factor 2, distance of 3 a factor of 3, and a distance of 1 a factor of 4. Finally, the cells where normalized by calculating the logarithm plus one. The singular value decomposition (SVD) algorithm was applied to the resulting matrix, where this algorithm compresses the information in the original matrix while maintaining as much information as possible. We call the resulting matrix a semantic representation of the words. A synonym test was used to find the optimal number of dimensions. This test was done by calculating the similarity score between two synonyms (as ranked by humans) and compares this score with randomly generated word pairs. This test was repeated using 1, 2, 4, 8, 16… etc. first dimensions in the semantic space. The highest score on this test was found using 256 dimensions, where the medium rank order of the semantic similarity between two synonyms was approximately 1.65% of the ranked order of semantic similarity of randomly generated pairs of words, where the semantic similarity was following the convention in the literature [32] measured as the cosines of the angle between the two vectors in the semantic representation. The result of this analysis is a semantic quantification of the 120000 most frequent words in the Swedish corpus, where each word is described by a 256 dimensional vector normalized to a length of one. Words that are semantically similar (e.g., synonyms) have similar representation in this semantic space.

Each participant's event narration was summarized in the semantic space by summing the semantic vectors representing each word in all narratives generated by that participant. Each word was weighted equally. The resulting vector was, similar to all word vectors in the space, normalized to a length of one.

Four semantic scales were generated from the experimental data along the axes spanning one condition relatively to the three other conditions. For example, a semantic scale of the visual conditions was conducted by first coding the visual condition as +1 and the three other conditions (auditory, olfactory, and multimodal) as −1. The semantic scales were then generated by predicting this coding using multiple-linear regression. A one-subject-leave out procedure was used, so that the-to-be predicted representation from one subject was removed from the training set (where the coefficient of the multiple linear regression is generated), and where these coefficients are applied to make a prediction on the left-out-subjects test representations. The predicted values on the test set constituted the semantic scale, where a positive value corresponds to semantic similarity to the predicted condition and a negative value to the three other conditions.

The semantic scale was first conducted separately for each word class (i.e., nouns, adjective, proper name, verb, adverb, participle, pronouns, conjunction, determiners counting-words, particle, prepositions, interpunction, and interjection). Then these semantic scales were combined, using the same one-subject-leave out multiple linear regression method as described above. This combined semantic scale was used to study the empirical data as described below.

The distribution of the data points on the semantic scale tends to be normally distributed (as measured by a Kolmogorov-Smirnov test) with mean around zero. Thus, a new training and testing was made for each participant. To avoid over-fitting, a subset of the dimensions in a semantic representation were used, where this subset was selected by using the most predictive dimensions in the training set, where the dimensions was ordered by how well they predicated the data on the training set. The number of dimensions used was set to one half of the total number of predicted data points. To evaluate whether the empirical values significantly predicts the semantic content, we correlated the empirical values with the predicted values. A significantly positive correlation indicates that the semantic representation predicts the outcome variable.

Each semantic scale generated a data point for each participant's aggregated narratives (n = 80), so that each participant's narrative was associated to four data-points associated to multimodal, visual, auditory and the olfactory semantic scales respectively. T-tests were used to establish whether the data points on the semantic scales differed significantly between conditions. One-sided t-tests were used to test the directed hypothesis whether the visual modality produced more positive values on the visual semantic scale compared to other conditions, and similar directed hypothesis were made on the other three semantic scales. Z–values were produced by first z–transforming the semantic scale score by using the mean and the standard deviation, and then dividing by the square root of the number of data points minus one. All analyses of the semantic representations were conducted using the Semantic program, which is a non-commercial software, written by Sverker Sikström, running in the MATLAB environment specially designed for analyzing and research on semantic representations. A web-based version of semantic will be available on http://saplo.silfverstrom.com/index.php/semantic/login.

## Results

### The differential hypothesis

**Figure 1:**
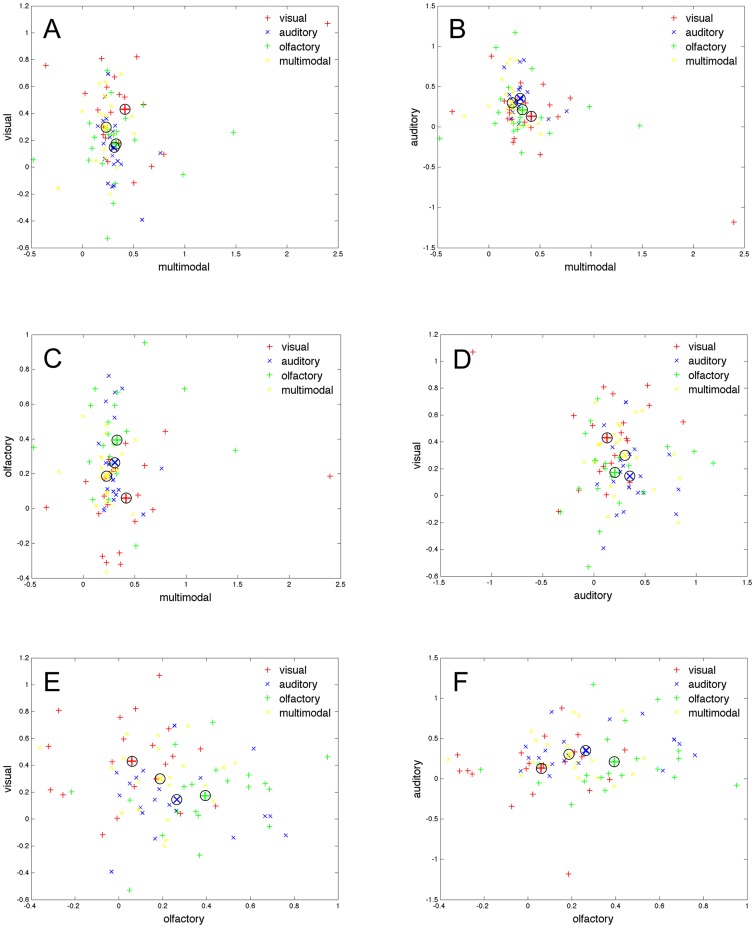
Data-points plotted for all pairwise combinations of the four semantic scales. *Note*. The axis represents the semantics scales (multimodal, visual, auditory, or olfactory). Panel A–F represent all possible pairwise combinations these scales, e.g., panel A have the multimodal scale on the x-axes and the visual semantic scale on the y-axis. The markers represent the participants' aggregated narratives on the semantic scale either as red crosses (visual condition), blue crosses (auditory condition), green crosses (olfactory condition), or yellow crosses (multimodal condition). See the methods section for details of how the semantic scales were computed. The four circles represent the mean value for the four conditions.

The visual, auditory, and olfactory semantic scales indicated that the unimodal conditions had semantic representations that were significantly different from the other conditions combined; for p-values see first column of Table 1, and for effect sizes (Cohen's *d*) see first column of Table 2. The multimodal scale indicated that the multimodal condition did not differ significantly from the three unimodal conditions combined. Figures 1A–F plot all data points along the semantic scales spanning one condition relative to three remaining conditions combined. For example, on the axis labeled “visual”, the semantic scale is generated from the visual condition (coded as +1 in the training dataset) relative to the combined data from the olfactory, auditory and multimodal conditions (coded as −1 in the training dataset). The fact that visual conditions tended to have more positive, and the olfactory, auditory, and multimodal conditions tended to have negative values on the semantic scale, indicate that semantic scales discriminate between the two groups of the conditions. Each condition is color coded, and the figures 1A–F correspond to all possible pairwise combinations of the axes.

**Table 1:** *P-values* showing differences between conditions (columns) on four semantic scale (rows).

Semantic scale	All other	Multi modal	Visual	Auditory	Olfactory
**Multimodal**	.921	–	.936	.955	.863
**Visual**	**.001**	.079	–	**.001**	**.005**
**Auditory**	**.050**	.261	**.019**	–	.070
**Olfactory**	**.001**	**.005**	**.001**	.062	–

*P-values* ≤.05 are highlighted in boldface. *Note*. The rows represent data from the four semantics scales (multimodal, visual, auditory, olfactory) respectively contrasted to the three other conditions. See the text for details of how to calculate the semantic scales. Each cell represents the *p*-values as calculated from t-tests. The first column compares one condition with three other conditions; the last four columns are pairwise comparisons between conditions. *P*-values were not corrected for multiple comparisons. Notice that the results are not symmetrical because each row represents different scales, thus, the olfactory value on the visual scale (row 2, column 6) differs from the visual value on the olfactory scale (row 4, column 4).

**Table 2:** Effect sizes (Cohen's *d*) for pairwise differences.

	All others	Multi modal	Visual	Auditory	Olfactory
**Multimodal**	−.368	–	−.491	−.549	−.352
**Visual**	.806	.457	–	1.012	.869
**Auditory**	.425	.205	.679		.476
**Olfactory**	.905	.866	1.357	.497	–

Note. See note in Table 1.

We further studied how each pairwise condition differed on the four semantic scales described above (see the last four columns in Table 1 and 2 for probabilities and effect sizes respectively). On the visual semantic scale, the visual condition differed significantly from the auditory and olfactory conditions, but differed only marginally (*p*<.10) from the multimodal condition. On the auditory scale, the auditory condition differed from the visual condition, but not from the multimodal condition. The difference between the auditory and the olfactory conditions was marginally significant (*p*<.10). On the olfactory scale, the olfactory condition differed from the visual and multimodal condition, and only marginally (*p*<.10) from the auditory condition. Finally, on the multimodal scale the multimodal condition did not differ significantly from any of the unimodal conditions (*p*s>.05). These results provided support for the differential hypothesis, which suggests that the semantic representation of events evoked by unimodal cues differ.

### The visual dominance hypothesis

**Figure 2:**
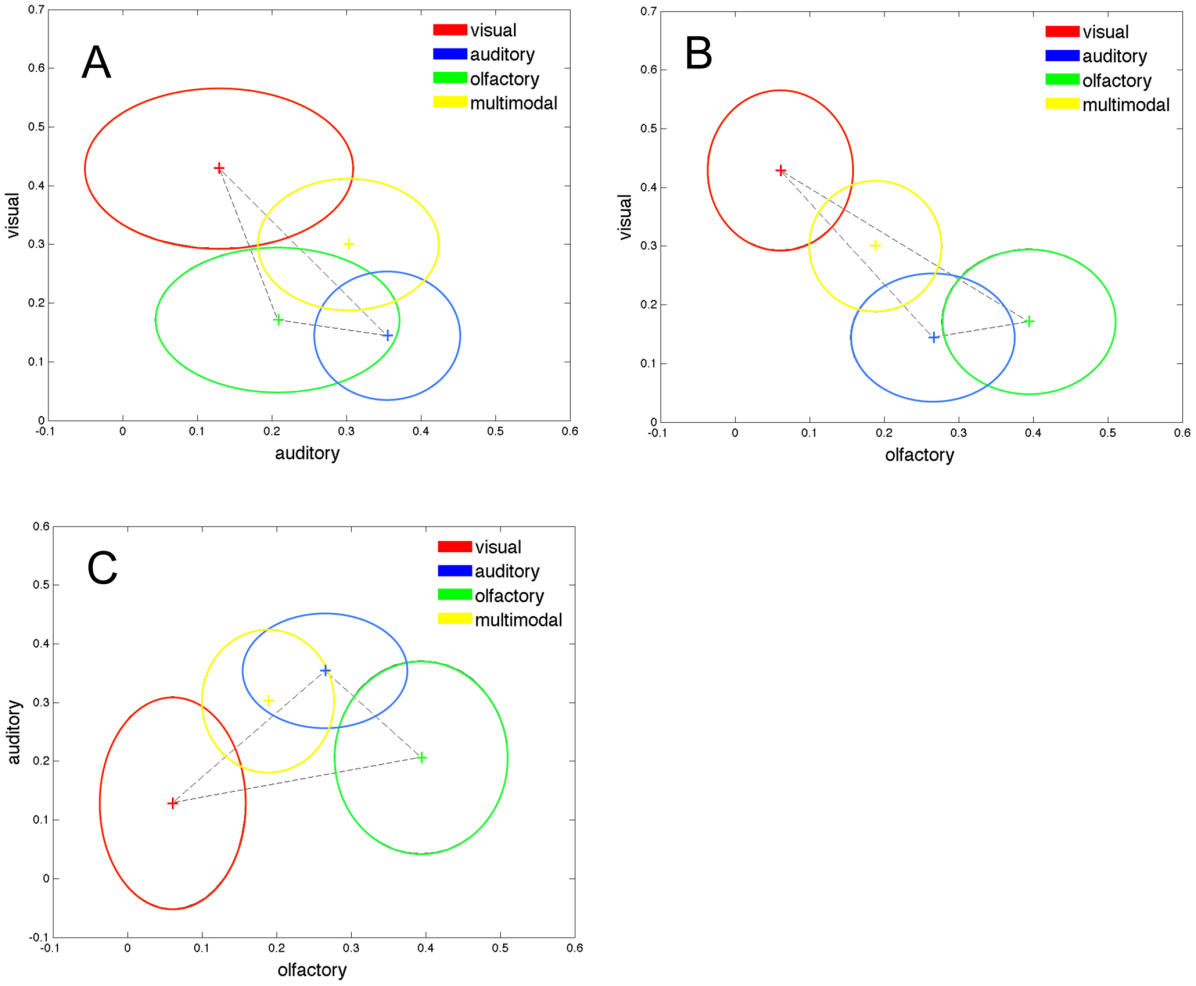
Mean and standard deviations of each condition plotted for the pairwise unimodal combinations of the four semantic scales. The axis represents the semantics scales (multimodal, visual, auditory, or olfactory). Panel A–C represent all possible pairwise combinations these scales, e.g., panel A have the auditory scale on the x-axes and the visual semantic scale on the y-axis. Crosses represent mean values for each condition (red crosses the visual-, blue crosses the auditory-, green crosses the olfactory-, and yellow crosses the multimodal condition respectively) and the circles represents a 95% confidence interval.

The visual dominance hypothesis was addressed by calculating effect sizes between each pair of the four conditions (see Table 2). A lower effect size between a unimodal condition and the multimodal condition indicates that the two conditions are closer located. The effect sizes in Table 2 indicate that the auditory and the multimodal condition were the closest (*d* = .205) followed by the visual and the multimodal (*d* = .457). The largest effect size was that between the olfactory and the multimodal conditions (*d* = .866). However, in addition to the effect sizes, inspection of Figures 2A–C suggests that both the auditory and visual conditions may be dominating.

### The triangular hypothesis

The triangular hypothesis was tested in the following way: 95% confidence intervals were calculated for each condition. An overlap between the unimodal triangle and the multimodal confidence interval would be indicative of the multimodal condition being located in the conceptual space spanned by the three unimodal conditions. That is, within this triangle the multimodal condition could be described as a linear combination of the unimodal conditions. Confidence intervals for the four conditions are presented in Figure 2A–C. The 95% confidence intervals indicate that there is a substantial overlap between the multimodal condition and the unimodal triangle (see Figure 2A–C). The analyses related to the differential hypothesis provide further support for the finding that the multimodal condition is confined within the unimodal triangle since the multimodal condition did not differ from the unimodal conditions combined. Thus the triangular hypothesis is supported in the present study.

## Discussion

The application of latent semantic analysis in the present study allowed us to quantify the semantic representation of a large data set by utilizing a computational algorithm. In the present study we applied this to the semantic representation of autobiographical events cued by unimodal and multimodal cues. The following three hypotheses were tested: the differential hypothesis, the visual dominance hypothesis, and the triangular hypothesis. Confirming the differential hypothesis suggests that events cued by visual, olfactory, auditory information differ in their semantic representations. That is, the content (meaning) of retrieved events differ as a function of cue modality. This is the first study to demonstrate such an effect. The visual-dominance hypothesis was supported to some extent suggesting that the semantic representations of auditory and visually evoked events are closer to multimodally evoked events than olfactory evoked events. Our results also provided support for the triangular hypothesis. The present results indicate that the multimodal representation may be regarded as a combination of the unimodal representations.

Given that previous research has observed differences in event recollections as a function of cue modality, it was of interest to study whether these differences would be reflected in the semantic representation of retrieved event information. Consistent with the differential hypothesis it was found that the semantic representation of event information was dependent on the cue modality. We argue that these results indicate that event information is selected and retrieved based on meaning. Several studies have suggested that autobiographical memories are temporary constructions built from information of an autobiographical knowledge base (e.g., [13]). Bits of information are searched for and selected during the retrieval process according to the specifications such as those suggested by Norman and Bobrow [17]. In the case of generative retrieval, when a retrieval cue is introduced a specification based on this cue is set up and information matching the specification is searched for [17]. Given that we observed significant differences in the semantic representation of the events across cue modality, the search and selection of information is not just a random process in relation to the cue modality. Consequently, a central question that arises concerns how these non-random content differences occur. We suggest these effects occur during retrieval and that meaning can play a role for both generative and direct retrieval.

With regard to generative retrieval, we suggest that the specification of information to be retrieved may be based on the semantic relationship with the retrieval cue. We also suggest that retrieval may occur when there is a semantic match between the specification and some information in the autobiographical knowledge base. If a specification and a particular piece information do not match with regard to their meanings the search continues in a cyclic fashion (see [17]). Meaning-based retrieval may not be limited to generative retrieval in our view. It could be argued that for direct retrieval, activation of event information based on semantic properties may occur in a similar fashion to that of an association. That is, the one-to-one mapping between a cue and some autobiographical information in the ESK could be based on meaning instead of a perceptual correspondence between the cue and the ESK information. It should be noted that the present design did not allow us to differentiate between the two forms of retrieval. In addition to the significant differences across cue modalities we also observed quite substantial effect sizes indicating that these effects are not trivial. It should be noted that we do not suggest that meaning-based retrieval is the only form of retrieval. Rather, what we suggest is that meaning-based retrieval is one of several mechanisms underlying retrieval of autobiographical events.

An alternative explanation for the present findings concerns encoding rather than retrieval. That is, there could be a systematic association of sensory modalities to specific types of events. For example, if a specific sensory modality is more perceptually salient compared to other modalities in some types of events during encoding, the likelihood of later retrieval for that type of events could potentially increase for events cued by that specific modality. Thus, according to this explanation the present results are driven by how sensory information is associated and integrated with autobiographical events at the level of encoding.

From an applied perspective the confirmation of the differential hypothesis has interesting implications. Our results indicate that the content of autobiographical memories differ depending on the retrieval cue. This may have implications for all domains that probe individuals' autobiographical events with perceptual cues. For example, if perceptual cues were to be adopted in a witness context it would be expected that the verbal statements of the witnesses would differ in content across cue modalities. Another domain for which the differential hypothesis may have critical implications is therapy. Given that the content of autobiographical memories differ depending on cue type, the presence of for example colors, odors, or sounds in a witness or clinical setting may influence what autobiographical information is being retrieved. For example, the perceptual constituents of an environment in which a therapy session takes place could potentially influence what information a patient retrieves. Thus, the present results stress the importance of how and potentially also under which environmental circumstances autobiographical information is probed. However, more research is needed to investigate exactly how the content of perceptually cued autobiographical events differs.

One of the main findings of the present study was that the distance between the cluster centroids for the four cue modalities followed a specific pattern that to some extent is consistent with the visual dominance hypothesis. That is, events retrieved with auditory and visual cues were located closer (in terms of effect sizes) to the multimodal events in the semantic space than olfactory cued events. In addition to the effect sizes, inspection of Figure 2A–C indicates that the visual condition plays an important role for the semantic representation of multimodally cued events. Since the present results are partially in agreement with the visual dominance hypothesis and we interpreted the results as follows. If the auditory and visual sensory systems are dominant in relation to other sensory systems and there is a modality hierarchy, it would be expected that event information retrieved with multimodal cues also reflects this hierarchy. This is because when all sensory systems contribute to the event retrieval and information selection to different degrees, the semantic representation of multimodally cued events should be closer to the semantic representation of the most dominant representation, second closest to the second most contributing and so on. Thus, we expect the distance between the semantic representation of multimodally cued events and the unimodally cued events to be a function of the relative contribution of the respective (uni)modality. To some extent this is the pattern we observed in the present data. Given the results of the present study in conjunction with the results of Willander, Sikström and Karlsson [unpublished data] it is suggested that both visual and auditory information may be dominant modalities.

With regard to Gärdenfors' [22] theory on conceptual spaces we found that the multimodal condition was mostly located inside of the triangles spanned by the unimodal conditions (see Figure 2A–C). We hypothesized that if the multimodal condition was located within the triangle then the multimodal condition could be described as a linear combination of the unimodal conditions. If however the multimodal condition was located outside of the triangle the multimodal condition could not be described by the unimodal conditions. Given that the multimodal condition was mostly located inside of the triangle and could therefore be described as a combination of the unimodal conditions, we argue that these results support the triangular hypothesis but does not support the notion of supra-additive effects (i.e., “the multisensory whole is greater than the sum of its unisensory parts”, [33] (p.R763) in multimodal retrieval of autobiographical events. Since supra-additive effects are common in multimodal integration (see, e.g., [33], [34]) it is interesting that the present study did not provide support for this notion in autobiographical event retrieval. Thus, multimodal retrieval cues trigger autobiographical information that to a large extent can be described by the unimodal conditions.

Taken together, the present study highlights the important interaction between perceptual and cognitive processing by addressing how different kinds of perceptual cues influence retrieval. From a theoretical point of view, the present study suggests that the pattern of activation in the self-memory system [13] could be driven by how information is related semantically. Furthermore, the present results may not be limited to autobiographical memory but could potentially apply to episodic memory retrieval in general. However, whether these results are valid for episodic memory in general need to be addressed in future studies.

In summary, the following was demonstrated in the present study: the content of retrieved events differ as a function of cue modality (the differential hypothesis); the semantic representations of auditory and visually evoked events where the closest to multimodally evoked events, followed by olfactory evoked events (the visual dominance hypothesis); the semantic representation of the multimodal condition could be described as a combination of the unimodal conditions (the triangular hypothesis). Overall, these results suggest that the meaning of the retrieved event information depends on the modality of the retrieval cues.

## Supporting Information

Appendix S1 **The stimuli of the four conditions.**
(DOCX)
