# Supplementary material for: The Semantic Representation of Event Information Depends on the Cue Modality: An Instance of Meaning-Based Retrieval
Source: PLoS One. 2013 Oct 28;8(10):e73378. doi: 10.1371/journal.pone.0073378 (PMC3810467; doi:10.1371/journal.pone.0073378)
Supplement: Appendix S1 — The stimuli of the four conditions. (DOCX) [file pone.0073378.s001.docx]

Appendix A

| **Visual** | **Auditory** | **Olfactory** |
| --- | --- | --- |
| Harbor with boats | Boat, waves, and seabirds | Fish |
| Pine forest | Walking in a forest, sticks breaking | Pine |
| Indoor swimming bath | Water splashes, laughter | Chlorine |
| Half-eaten apple | Biting and chewing an apple | Apple |
| Washing machine | Washing machine in use | Liquid washing detergent |
| Mop and bucket | Mopping a floor, mop in a bucket with water | Soft soap |
| Bar table with a glass of beer | Typical bar sounds (no music) | Beer |
| Café table with a cup of coffee | Typical café sounds (no music) | Coffee |
| Car at a gas station | A car being filled with gas | Gasoline |
| Cigarette and ashtray | Lightning a cigarette inhale/exhale sounds | Cigarette |
| Garden with flowers | Bees, ambient outdoor sounds | Flower |
| Onions being chopped | Chopping onions | Onion |
| Campfire | The sound of burning wood (campfire) | The smell of burnt |
| Wood panels being painted | Opening a jar of paint, brush strokes | Wall paint |
| A dentist with a drill | Dentist drill and saliva suction | Eugenol |
